# Oceanic Crustal Fluid Single Cell Genomics Complements Metagenomic and Metatranscriptomic Surveys With Orders of Magnitude Less Sample Volume

**DOI:** 10.3389/fmicb.2021.738231

**Published:** 2022-01-24

**Authors:** Timothy D’Angelo, Jacqueline Goordial, Nicole J. Poulton, Lauren Seyler, Julie A. Huber, Ramunas Stepanauskas, Beth N. Orcutt

**Affiliations:** ^1^Bigelow Laboratory for Ocean Sciences, East Boothbay, ME, United States; ^2^School of Environmental Sciences, University of Guelph, Guelph, ON, Canada; ^3^School of Natural Science and Mathematics, Stockton University, Galloway, NJ, United States; ^4^Marine Chemistry and Geochemistry, Woods Hole Oceanographic Institution, Woods Hole, MA, United States

**Keywords:** deep biosphere, oceanic crust, crustal fluid, single cell genomics, metatranscriptomics, IODP, CORKS, North Pond

## Abstract

Fluids circulating through oceanic crust play important roles in global biogeochemical cycling mediated by their microbial inhabitants, but studying these sites is challenged by sampling logistics and low biomass. Borehole observatories installed at the North Pond study site on the western flank of the Mid-Atlantic Ridge have enabled investigation of the microbial biosphere in cold, oxygenated basaltic oceanic crust. Here we test a methodology that applies redox-sensitive fluorescent molecules for flow cytometric sorting of cells for single cell genomic sequencing from small volumes of low biomass (approximately 10^3^ cells ml^–1^) crustal fluid. We compare the resulting genomic data to a recently published paired metagenomic and metatranscriptomic analysis from the same site. Even with low coverage genome sequencing, sorting cells from less than one milliliter of crustal fluid results in similar interpretation of dominant taxa and functional profiles as compared to ‘omics analysis that typically filter orders of magnitude more fluid volume. The diverse community dominated by Gammaproteobacteria, Bacteroidetes, Desulfobacterota, Alphaproteobacteria, and Zetaproteobacteria, had evidence of autotrophy and heterotrophy, a variety of nitrogen and sulfur cycling metabolisms, and motility. Together, results indicate fluorescence activated cell sorting methodology is a powerful addition to the toolbox for the study of low biomass systems or at sites where only small sample volumes are available for analysis.

## Introduction

Oceanic crustal fluids may be significant habitats for life on Earth, yet relatively little is known about the composition of life in these habitats, and even less is known about the functional potential of their microbial communities (Orcutt B. et al., 2020). Understanding life in these fluids is important as it can significantly impact global chemical cycling (Orcutt et al., 2013; Shah Walter et al., 2018; D’Hondt et al., 2019; Wheat et al., 2020; Trembath-Reichert et al., 2021). On the ridge flanks, direct sampling of crustal fluids is possible with borehole observatories that penetrate through sediment and oceanic crust. Currently there are two focal sites for examining life in subsurface crustal fluids on ridge flanks: one in the cold (4–25°C) and oxic basaltic subsurface of the western flank of the Mid-Atlantic Ridge, and the other in the warm (∼65°C) and anoxic subsurface of the eastern flank of the Juan de Fuca Ridge (Edwards et al., 2012a; Orcutt B. et al., 2020; Orcutt B. N. et al., 2020).

The microbial communities that inhabit circulating fluids at these sites are low biomass with cell densities of 10^2^–10^4^ cells ml^–1^ (Jungbluth et al., 2013; Nigro et al., 2017; Tully et al., 2018; Orcutt B. N. et al., 2020; Trembath-Reichert et al., 2021), which makes them challenging to study. Large volumes (10^1^–10^4^ L) of crustal fluids are often required to obtain sufficient biomass for sequencing nucleic acids (i.e., DNA or RNA) to determine community structure, functional potential, and transcriptomic activity using current metagenomic and metatranscriptomic technologies. Collecting these large volumes requires *in situ* fluid pumping systems (Cowen et al., 2012; Li et al., 2020) that also necessitate considerable payload and time on the seafloor with remotely operated vehicles (ROVs) for sampling. Considering the infrequency of expeditions along with the above sampling limitations, only a handful of metagenomic datasets have been reported from these environments, and only one metatranscriptomic dataset has been reported (Jungbluth et al., 2017; Tully et al., 2018; Seyler et al., 2020).

The one metatranscriptome dataset from the basaltic subsurface of the Mid-Atlantic Ridge—a site called North Pond (Edwards et al., 2012a; Orcutt et al., 2013; Wheat et al., 2020)—revealed a motile and mixotrophic microbial community fueled by oxygen, nitrate, and thiosulfate energy sources (Seyler et al., 2020). Evidence for autotrophic metabolisms was most strongly associated with sulfur, iron and nitrogen cycling taxa. Ammonium oxidation in this habitat is attributed to low abundance *Nitrosopumilaceae* Archaea, as has been observed in other studies in this and similar systems (Zhao et al., 2020), while there is no evidence for active nitrogen fixation in this habitat. This functional interpretation is based on mapping metatranscriptome libraries to 64 high-quality metagenomic bins from a study of ten samples collected across multiple years and holes at North Pond in addition to mapping reads to individual annotated genes from assembled, un-binned metagenomic sequences. Moreover, these metatranscriptomes were dominated by ribosomal RNA as compared to messenger RNA, since ribosomal material was not removed due to the low biomass of the samples (Seyler et al., 2020).

In this study, we sought to evaluate the utility of a new approach for examining the microorganisms in environmental samples through single-cell sorting using a redox active dye. This approach is based on the incorporation of oxidoreductase-sensitive RedoxSensor*™* Green (RSG) into cells (Kalyuzhnaya et al., 2008; Konopka et al., 2011; Beam et al., 2020; Becraft et al., 2021) followed by indexed fluorescence-activated single cell sorting with a flow cytometer (Stepanauskas et al., 2017) to enable generation of single cell amplified genomes. A major advantage of this approach is the ability to create hundreds of individual microbial genomes from even just one milliliter of a low-biomass fluid sample (Stepanauskas et al., 2017)—5–8 orders of magnitude less volume than has previously been used for either metagenomic or metatranscriptomic approaches from low biomass crustal environments. By basing the sorting on only cells with active oxidoreductase function, the resulting dataset also provides a means to link functional phenome information to specific genomes (Beam et al., 2020; Becraft et al., 2021), although further benchmarking of this approach is required. Here, we evaluated this single cell approach with one crustal fluid sample that was subsampled from the same crustal fluid described in the recent metatranscriptomic study from the North Pond location (Seyler et al., 2020). By comparison to the Seyler et al. (2020) datasets, we demonstrate that similar taxonomic and functional profiles result from these two approaches, even with low coverage single cell genomic sequencing.

## Materials and Methods

### Sampling and Addition of RedoxSensor Green

The sample used in this study was collected in October 2017 during cruise AT39-01 on R/V *Atlantis* using ROV *Jason II* to access the subseafloor borehole observatories previously installed at North Pond by the Integrated Ocean Drilling Program (IODP; Edwards et al., 2012b). As described in other recent studies (Seyler et al., 2020; Trembath-Reichert et al., 2021), subsurface crustal fluid from the “deep” horizon of the observatory in IODP Hole U1383C, sampling fluid from 200 to 332 m below seafloor, was collected into a sterile bag using the Mobile Pumping System (Cowen et al., 2012; Lin et al., 2020). A 10 mL aliquot of this Hole U1383C Deep fluid was subsampled shipboard for this study under an UV-irradiated, HEPA-filtered hood, with the remainder of fluids used for other analyses (Trembath-Reichert et al., 2021). The sub-sampling was not performed until approximately 10 h after the original sample collection on the seafloor. The fluid sat in the sample collection bag on the chassis of the ROV for approximately 5 h on the seafloor, prior to a 3-h transit to the surface. Upon being brought on board the ship, the sub-sampling and proceeding sample treatment was performed immediately, with incubation with RedoxSensor*™* Green (RSG) beginning within 2 h. RSG reagent from the BacLight*™* Vitality Kit (Invitrogen/Thermo-Fisher Scientific part B34954) was added in a 1:1,000 ratio to sample in 2 mL cryovials per manufacturer instructions. Five 1-mL aliquots of sample with RSG were incubated in the dark at 4°C for 40 min, followed by addition of 100 μl of 10 × Glycerol-Tris-EDTA buffer [55% (v/v) molecular-grade glycerol, 33.3% (v/v) deionized water, 13.3% (v/v) 100 × Tris-EDTA pH 8.0] and flash frozen with liquid nitrogen. After shipment to the shore-based laboratory under frozen conditions, samples were stored in an ultracold freezer until analysis approximately 2 months later. In addition to the one sample analyzed in this study, parallel 16S rRNA gene amplicon, metagenomic and metatranscriptomics analysis of crustal fluids from the Hole U1383C observatory, as well as other fluid samples, has been described elsewhere (Seyler et al., 2020; Trembath-Reichert et al., 2021).

### Single Cell Sorting and Sequencing

At the Single Cell Genomics Center at the Bigelow Laboratory for Ocean Sciences, one RSG subsample was thawed and gently vortexed, then passed through a 40-μm-mesh nylon filter in preparation for fluorescence activated cell sorting following protocols described previously (Stepanauskas et al., 2017). Cell-like particles were sorted in a clean room on a Becton Dickinson Influx Mariner cytometer (formerly Cytopeia) with sterile 15 ppt NaCl solution as sheath fluid following excitation with a 488 nm wavelength blue solid-state laser. The detection window (gate) for cells that incorporated RSG was chosen manually based on red (emission filter: 692 nm wavelength with 40 nm bandpass) and green (emission filter: 531 nm wavelength with 40 nm bandpass) relative fluorescence. Individual cell-like particles with RSG fluorescence were sorted into individual wells of a 384-well plate followed by lysis and multiple displacement amplification of DNA with the WGA-X DNA polymerase as described previously (Stepanauskas et al., 2017) to create single cell amplified genomes (SAGs). There was no pre-screening or selection of which wells to sequence, except for a successful WGA reaction, as described in Stepanauskas et al., 2017. Low-coverage sequencing of SAGs, genome assembly and gene-calling were performed by the standard SCGC Low Coverage (LoCos) sequencing protocol previously described (Stepanauskas et al., 2017).

### Analysis of Single Cell Genomes and Comparison to Previously Published Data

To compare the single cell genomes with bulk ‘omics approaches, the SAG assemblies were used to recruit reads from previously-published metagenomic and metatranscriptomic datasets (Tully et al., 2018; Seyler et al., 2020). Whereas the SAG dataset originated from only the Hole U1383C Deep fluids sampled in 2017, our comparison paired metagenomic and metatranscriptomic datasets sourced from all depth horizons of Hole U1383C and the neighboring observatory at Hole U1382A sampled in 2012, 2014, and 2017 (Tully et al., 2018; Seyler et al., 2020).

A total of 447 metagenome assembled genomes (MAGs) were obtained from the 2017 North Pond metagenomes. Seyler et al. (2020) focused on analyzing 64 non-redundant, high-quality, high-completion bins. Here, we include the 131 “high-completion” and “low-completion” MAGs from the Hole U1383C Deep horizon from the total 447 that were sampled. Note that the terms “high-completion” and “low-completion” described in Seyler et al. (2020) are not synonymous with commonly used thresholds for high- and low-quality genomes (Parks et al., 2015; Bowers et al., 2017), and “low-completion” MAGs were not discussed in Seyler et al. (2020). Here, as in Seyler et al., high-completion is defined as > 90% complete with < 10% redundancy, greater than 80% with < 5% redundancy, or > 50% with < 2% redundancy, and low-completion is defined as < 50% complete with < 5% redundancy. Average Nucleotide Identity (ANI) between the SAGs and the 131 MAGs were computed using FastANI using the SAGs and MAGs as both the reference and query datasets (Jain et al., 2018). A fragment length of > 3,000 bp was used in ANI computation (FastANI default). SAG to MAG ANI matches were only considered if > 75% of the SAGs > 3,000 bp fragments mapped to a MAG and the corresponding reverse MAG to SAG comparison yielded a similar ANI score.

Metagenomic and metatranscriptomic reads were quality controlled and mapped to the SAGs at a 95% or greater sequence identity following the same protocol as Seyler et al. (2020). Briefly, trimmomatic was used to trim raw reads using the following parameters: SLIDINGWINDOW:10:28 MINLEN:75. Only reads pairs retaining both forward and reverse reads after trimming were used (Bolger et al., 2014). Reads were mapped to the SAG library using Bowtie2 (using the –no-unal flag) and resulting sam files were converted to bam format and sorted using Samtools (Li et al., 2009; Langmead and Salzberg, 2012). BamM was used to filter reads from the bam files using the parameters: --percentage_id 0.95 --percentage_aln 0.75^1^. BinSanity was used to make coverage profiles for the SAGs from the bam files, using the command Binsanity-profile, which were used to calculate coverage as Reads per Thousand Million (RPKM) for individual SAGs (Graham et al., 2017). Recruitment of metagenomic and metatranscriptomic reads per sample were calculated as the percentage of the read-pairs that mapped to the SAG library. To determine the approximate rRNA/mRNA composition of the metatranscriptome libraries, they were mapped to the SILVA LSU/SSU databases (v138) (Quast et al., 2012). Read mapping was performed as described above, but reads mapped to the SILVA databases were not filtered on > 95% identity. Approximate composition of the libraries was determined by counting the percentage of reads that mapped or did not map to the SILVA databases.

Transcript Per Million (TPM) statistics for protein-coding genes in the SAGs were calculated by taking the metatranscriptomic reads that did not map to the SILVA LSU/SSU databases, or ribosomal genes in the SAGs, but that did map to the SAGs at > 95% and re-mapping them using Kallisto (Bray et al., 2016). The Kalliston -quant workflow, which accounts for both gene length and sequencing depth was utilized, using default parameters, in the same fashion that was performed in Seyler et al. (2020).

The North Pond Hole U1383C Deep SAGs and the high-completion and low-completion MAGs were clustered into approximate orthologous gene clusters. The nucleotide sequences were translated into protein sequences using Prodigal and an all-against-all Basic Local Alignment Search Tool (BLAST) BlastP protein search was performed using Diamond (Hyatt et al., 2010; Buchfink et al., 2015). Markov-chain clustering was performed on the results to form gene clusters, with weak connections being filtered by the MINBIT approach (Enright et al., 2002; Benedict et al., 2014). These processes were automated using the script “anvi-pan-genome” in the Anvio package (parameters –minbit 0.5 –mcl-inflation 2; Delmont and Eren, 2018; Eren et al., 2021). In order to determine if a plateau in new gene-clusters occurs in relationship to sampling effort—which would indicate the sampling effort effectively captured the functional diversity of the samples, this workflow was also performed on the SAG dataset alone, the high-completion MAGs alone, the low-completion MAGs alone, all MAGs combined, the SAGs combined with the high-completion MAGs, and the SAGs combined with all MAGs.

Functional annotations were performed on the translated SAG sequences using KOFAMSCAN (-mapper option; Aramaki et al., 2020). Comparison of gene presence and copy number of a set of functions-of-interest in the SAG dataset were compared to transcript per million (TPM) data for the set of genes in the metatranscriptome data from Seyler et al. (2020). Although these datasets are normalized in different ways, this comparison was done to qualitatively assess relative patterns between the two. KEGG decoder was used to query the KOFAMSCAN annotations for additional genes of interest (Graham et al., 2018).

A phylogeny from aligned conserved protein sequences from Zetaproteobacteria SAGs/MAGs and publicly available Zetaproteobacteria genomes downloaded from Integrated Microbial Genomes (IMG) was constructed using the standard protocol of PhyloPhlan (Asnicar et al., 2020). The Amphora2 marker-gene set was utilized with a minimum of one marker gene (Wu and Scott, 2012). A phylogenetic tree was created and annotated using the Interactive Tree of Life (iTOL) online tool (Letunic and Bork, 2019).

## Results

### Similarities Between SAG Assemblies and MAG Dataset

**FIGURE 1:**
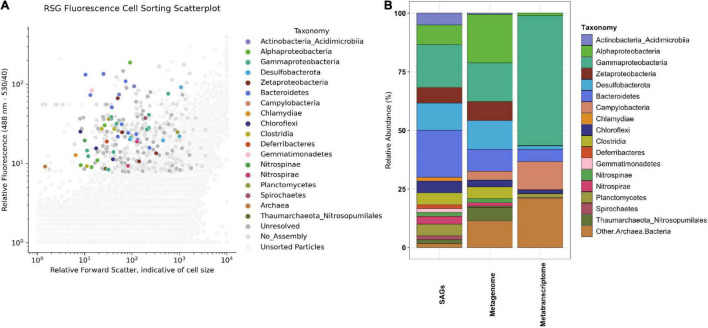
**(A)** Taxonomic identity of active single cells (i.e., sorted based on RedoxSensor*™* Green fluorescence) from subsurface crustal fluids from the deep horizon of IODP Hole U1383C collected in 2017 after indexed fluorescence activated cell sorting. The x-axis plots the particle/cell forward light scatter (FSC) after illumination with the 488 nm wavelength laser, indicative of cell size. The y-axis plots the relative fluorescence emission of particles (530 nm wavelength filter set with 40 nm bandpass). Color of symbol represents taxonomic identity of single cell amplified genome (SAG) according to legend, with dark gray symbols representing SAGs whose identity could not be resolved and lighter gray symbols indicating particles sorted that did not result in an assembled SAG. Small gray dots represent particles that were not index sorted for genome amplification. **(B)** Comparison of the relative abundance of taxa (at Phylum/Class level) in various sample types from North Pond Hole U1383C Deep fluids collected in 2017. The SAGs from this paper are the first column from the left, presented as the relative abundance of the 60 SAGs that received a taxonomic assignment *via* CheckM or BlastN of the 16S rRNA gene. The next two columns summarize the taxonomy from metatranscriptome and metagenome data from the 2017 U1383C Deep horizon described in Seyler et al. (2020).

Sorting cell-like particles from the sample using RSG fluorescence resulted in 131 of the 384 wells of the plate yielding successful amplification of single cell genomes (Figure 1A). Following low coverage genome sequencing (Stepanauskas et al., 2017), the 131 SAG assemblies ranged from 38 KB to 1.4 MB in length, with an average of 408 KB and a total length of 42.5 Mbp (see Supplementary Data Sheets 1, 2). Estimated completion of the low coverage SAGs determined by CheckM (Parks et al., 2015) ranges from 0–58% with an average of 9% completion (Supplementary Figure 1). These SAGs fall into the low-quality standards (<50% complete), according to the MIMAG/MISAG guidelines (Bowers et al., 2017), except for one medium-quality SAG. Of these 131 SAGs, 15 contained the 16S rRNA gene. For comparison, the combined 131 high-completion and low-completion MAGs from 2017 Hole U1383C Deep from Seyler et al. (2020) have an average length of 224 KB (range of 11.5KB to 3.45 MB, total length 84.9 MB) and average completion of 13.2% (range 0–99%; Supplementary Figure 1). The ten high-completion MAGs from Hole U1383C Deep have an average completion of 78% and a total length of 26.6 Mbp. All SAGs and MAGs had < 5% contamination, with an average of 0.13%.

SAG taxonomy was similar to the taxonomic assignment for MAGs (Figure 1B) and included Bacteroidetes (*n* = 12 SAGs), Desulfobacterota (*n* = 7), Zetaproteobacteria (*n* = 4), Actinobacteria (Class Acidimicrobiia; *n* = 3), and Clostridia (*n* = 3). Eighteen SAGs from Hole U1383C Deep 2017 sample share > 95% ANI with four of the eleven high-completion MAGs from the 2017 Hole U1383C Deep metagenome from Seyler et al. (2020). Furthermore, thirteen of these 18 SAGs also share > 95% ANI with eight MAGs from 2017 metagenomes from the Hole U1383C shallow and middle observatory depths (Supplementary Data Sheet 1). The Acidimicrobiia SAGs share ∼99% ANI with high-completion MAG NP171383CD-5 and the Desulfobacterota SAGs share ∼99% ANI with MAG NP171383CD-8.

### Read Recruitment to SAGs From Metagenomic and Metatranscriptomic Libraries

**FIGURE 2:**
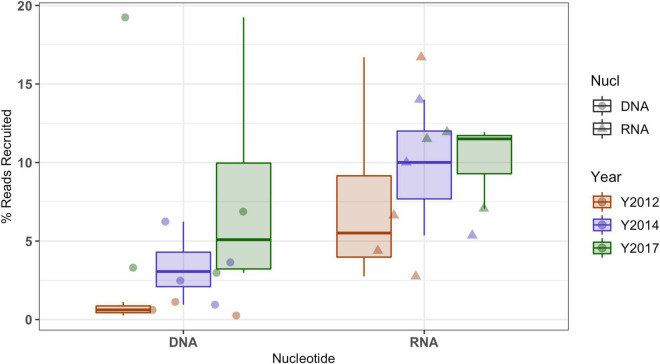
Recruitment of metagenomic (i.e., DNA) and metatranscriptomics (i.e., RNA) sequence reads from all North Pond Hole U1382A and Hole U1383C horizon fluid samples (i.e., from Seyler et al., 2020) from multiple years (indicated by color per legend) to low-coverage single cell amplified genomes (SAGs) from Hole U1383C Deep horizon collected in 2017. Mapped reads were filtered to those with > 95% ID to SAG sequences. Metatranscriptomic data includes both mRNA and rRNA mapping to ribosomal genes. The boxes represent the inter-quartile range (25th–75th quartiles) and the horizontal line inside the box is the median.

Mapping metagenome and metatranscriptome reads from all North Pond locations and horizons for all timepoints (2012, 2014, and 2017) to the 2017 Hole U1383C Deep low coverage SAGs shows two different trends (Figure 2 and Supplementary Data Sheets 3, 4). First, there is an increased percentage of metagenomic reads mapped to the SAGs over time, with the lowest in 2012 and highest in 2017. The average percentage of metagenome read recruitment from all 2012 samples to the 2017 SAGs is < 1%, which increases to 8% when comparing 2017 metagenomes to the 2017 SAGs, although the increase in read recruitment percentage is not statistically significant (Students *T*-test *p* > 0.05, data grouped by year). Within this, roughly 7% of the 2017 Hole U1383C Deep horizon metagenome recruits to the 2017 SAGs.

The percentage of metatranscriptomic reads from multiple years mapping to the 2017 SAGs appears less variable over time (average 9%). This metatranscriptome mapping includes both mRNA and rRNA. Mapping metatranscriptome reads from all years and locations to the SILVA LSU/SSU databases indicated that, on average only 6.7% of these libraries are mRNA (Supplementary Data Sheet 3). For 2017 Hole U1383C Deep, 13% of the metatranscriptome did not map to the SILVA databases, and is inferred to be mRNA. The SAG contigs recruited 7% of this metatranscriptome library from the Hole U1383C Deep 2017 metatranscriptome. Of these mapped reads, 93% recruited to the 15 SAGS with ribosomal operons, where 53% mapped to the 16S rRNA gene and the remainder to the other rRNA subunit genes on those contigs (Supplementary Data Sheet 5). The metatranscriptome reads that mapped to protein coding sequences represented only 0.1 % of the library and mapped to four SAGs with resolved taxonomy: SAG AH-315-J17, a member of the Zetaproteobacteria, and SAG AH-315-C21, a Gammaproteobacteria that is closely related to MAG NP171383CD-7, SAG AH-315-F22 a Bacteroidetes and SAG AH-315-K04 a Gammaproteobacteria (Supplementary Data Sheet 3).

**FIGURE 3:**
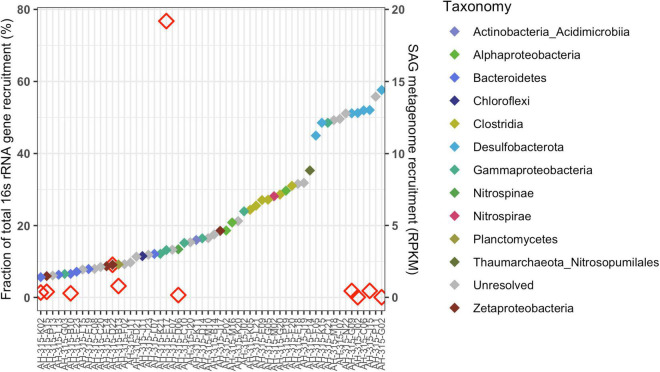
Recruitment of metagenomic (DNA) reads from the 2017 Hole U1383C Deep sample to the activity-based low-coverage SAGs from this same sample (displaying the SAGs having above-mean RPKM values, *n* = 58), sorted by SAG name (x-axis) from left to right in order of increasing recruitment value in reads per thousand million (RPKM) shown with colored filled diamonds according to the right y-axis. Symbol color indicates the taxonomy of the SAG at Class level per the legend. Fifteen of these low-coverage SAGs contained ribosomal genes, indicated by the open red diamonds. The fraction of total metatranscriptomic reads that mapped to 16S rRNA genes in these 15 SAGs is indicated on the left y-axis. Of note, all of the seven *Desulfobacterota* SAGs had the highest recruitment coverage, followed by SAGs from *Gammaproteobacteria, Thaumarchaeota, Clostridia*, *Alphaproteobacteria*, *Nitrospira*, and some unresolved.

Mapping metagenomic reads from 2017 Hole U1383C Deep and calculating reads-per-thousand-million (RPKM) coverage values for the individual SAGs largely replicates the taxonomic compositions of the SAG library and metagenomic library (Figures 1, 3). SAGs in Desulfobacterota, Bacteroidetes, Clostridia, Alphaproteobacteria, Gammaproteobacteria and Zetaproteobacteria have among the highest RPKM values, similar to their abundance in the metagenomic library (Figures 1B, 3). Since the read mapping analysis only included mapped reads with > 95% identity to the SAGs, these data indicate that very closely related cells to the SAGs are present in the metagenomic sample (Figures 1B, 3).

### Clustering Protein Coding Sequences

**FIGURE 4:**
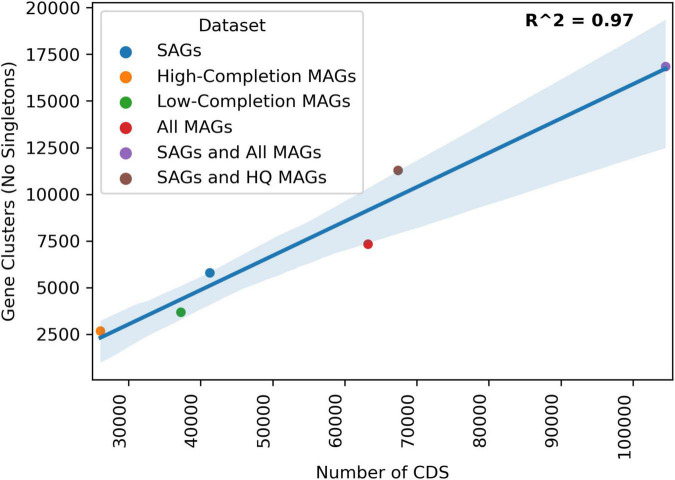
Linear regression of total Number of CDS in a given dataset to the amount of gene clusters produced by the Anvio Pangenomics workflow. HIGH-COMPLETION MAGs are the 10 high-completion MAGs discussed in Seyler et al. (2020). LOW-COMPLETION MAGs are the low-completion MAGs not discussed in Seyler et al. (2020) but produced from that same sampling, All MAGs are those the HIGH-COMPLETION and LOW-COMPLETION MAGs combined. The SAGs from this study were clustered alone (blue) and then added to the HIGH-COMPLETION MAGs (brown) or all of the MAGs (red).

Clustering protein coding sequences from both the SAG and MAG datasets from 2017 Hole U1383C Deep into gene clusters indicates that these datasets contain similar amounts of genetic information, linearly correlated to sequencing depth of the dataset (Figure 4). Individually, the SAGs, high-completion MAGs, and all MAGs produced 5,788, 2,671, and 7,324 non-singleton gene-clusters, respectively. The SAG dataset had an intermediate amount of genetic information, more than the high-completion MAGs but less than the total MAG datasets. Plotting the cumulative number of protein coding sequences (CDS) for the SAGs, high-completion MAGs, low-completion MAGs, all MAGs combined, and the SAG and MAG datasets combined, against the number of gene-clusters produced from respective datasets revealed a strong linear relationship (*R*^2^ = 0.97 for non-singleton gene clusters). Between the 131 SAGs and the 11 high-completion MAGs, there were 67,318 protein coding sequences resulting in 11,276 non-singleton gene-clusters (see Supplementary Data Sheet 6). Approximately 68% of these clusters contained sequences from both the SAGs and the MAGs, with an additional 24% of the gene clusters unique to the SAGs and approximately 9% unique to the high-completion MAGs. Between the 131 SAGs and the combined 131 high-completion and low-completion MAGs, there were 104,678 protein coding sequences clustered into 16,838 non-singleton gene-clusters, of which approximately 67% were shared, 10% were unique to the SAGs, and 23% were unique to the MAGs. The data reported in Seyler et al. (2020) also included gene-based analysis of un-binned assembled metagenome data, but the comparison here only includes assembled data that was included in a MAG.

### Functional Comparison Between Datasets

**FIGURE 5:**
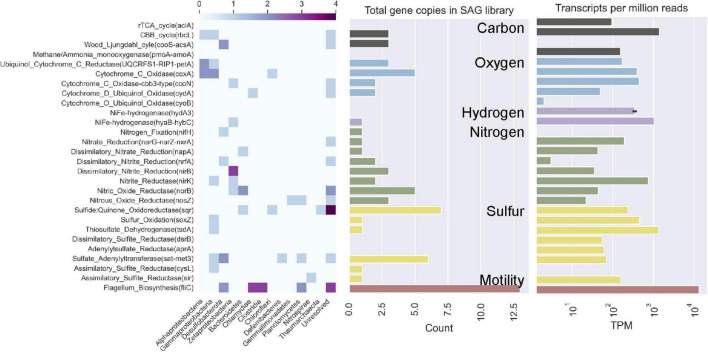
Comparison of functional potential of Hole U1383C Deep microbial community based on single cell amplified genomes (SAGs, this study) versus metatranscriptomic interpretation (from Seyler et al., 2020). Abundance of key genes for carbon, oxygen, hydrogen, nitrogen, and sulfur metabolisms and flagellum biosynthesis are shown on y-axis, in same order as presented in Seyler et al. (2020). In left panel, relative abundance of these genes in SAGs of various taxonomic groups are shown according to the heat map above. In the center panel, the total copies of these key genes in the entire low-coverage SAG dataset is shown for comparison to the metatranscriptomic observation shown in the right panel (as transcripts per million reads on a log scale, as reported in Seyler et al., 2020).

**FIGURE 6:**
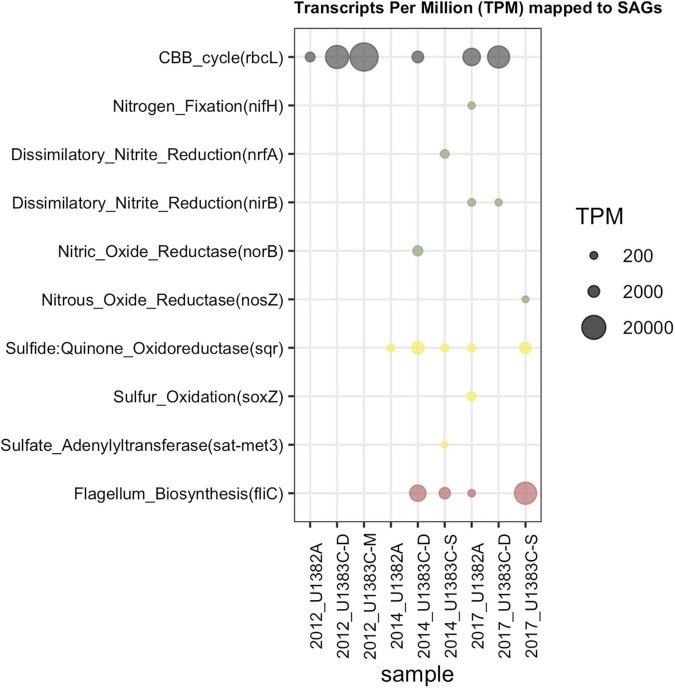
Transcript Per Million (TPM) data after mapping metatranscriptomes from all years and horizons to the SAG library from 2017 Hole U1383C Deep. The Y Axis lists the subset of key functions (as discussed in Seyler et al., 2020; Figure 5) from the 2017 Hole U1383C Deep SAGs that received mapped reads.

The functions encoded in the SAGs show high overlap with the functions of interest analyzed in Seyler et al. (2020) (Figures 5, 6). Mapping all metatranscriptome libraries from all years to protein-coding sequences from all SAGs resulted in 68 of the 131 SAGs receiving mapped metatranscriptome reads, although only in small amounts. A subset of the functions-of-interest discussed in Seyler et al. (2020) were present in these SAGs and received mapped metatranscriptome reads at > 95% identity (Figure 6). When combining all metatranscript reads, the patterns of relative abundance of reads mapped to SAGs was similar to that observed in the Seyler et al. (2020) study (Figure 5). For example, a key gene for flagella biosynthesis was more abundant than the other key genes in both SAG and metatranscriptome datasets.

Both datasets indicate the use of the Calvin-Benson-Bassham (CBB) cycle for carbon fixation, cbb-3 type cytochromes, and several pathways for nitrogen and sulfur cycling (Figures 5, 6). The CBB cycle gene *rbcL* in the SAGs mapped reads from several metatranscriptome libraries (Figure 6). The SAG dataset did not have evidence for the reverse tricarboxylic acid (rTCA) for carbon fixation nor evidence for hydrogenases, as was observed in the metatranscriptomes. Desulfobacterota SAGs contained nitrogen fixation genes of which SAG AH-315-G02 contained a *nifH* gene that mapped metatranscriptome reads from sample 2017 U1382A. Zetaproteobacteria SAG AH-315-E14 contains a putative nitrite reduction operon and nitric oxide reductase [*norB*, KEGG Orthology function identification (KO ID) K04561] mapped metatranscriptome reads from the 2014 Hole U1383C Deep library. SAGs also contained genes for sulfur oxidation (*soxZ*, KO ID: K17227) and thiosulfate dehydrogenase (*tsdA*, KO ID: K19713), respectively (Figure 5).

## Discussion

Understanding the activity and physiology of environmental microorganisms is a challenging pursuit (Widder et al., 2016), especially in low biomass environments. Next generation physiology approaches are proposed as possible mechanisms to address these challenges (Hatzenpichler et al., 2020), but few have been tested in low biomass settings. In this study, we evaluated the next-gen physiology approach of sorting of cells after short incubation with RedoxSensor*™* Green cells from a low biomass (∼10^3^ cells ml^–1^) deep biosphere crustal fluid (from Hole U1383C Deep collected in 2017), and compared the resulting interpretation of taxonomy and function of cells sorted with the redox-active dye with bulk metagenomes and metatranscriptomes from the same location sampled across multiple years (Seyler et al., 2020). The overall taxonomy, average nucleotide identity (ANI) values, protein-clustering and read mapping statistics between the sorting and sequencing of single cell amplified genomes (SAGs) and metagenomic and metatranscriptomic approaches show that both approaches lead to similar conclusions for low-biomass crustal fluid samples, even though the SAGs were only sequenced at low-coverage (average 9% genome completeness; Figures 1, 3, 5, 6). For example, protein-coding statistics show that there is a large amount of overlap in the functional information that is captured in the SAG and MAG datasets, with two-thirds of identified gene clusters containing sequences from both datasets (Figure 4 and Supplementary Data Sheet 6). Further, the fact that 16/131 (12.2%) of the SAGs have a high ANI match with a MAG indicates high overlap in the datasets, even though the MAGs range from 50 to 99% (average 78%) completion and the SAG completion is on average 9%.

### Comparison of Observations Between Methodologies

The metagenomic and metatranscriptomic datasets from 2017 were created from *in situ* filtered samples that required active pumping and filtering of ∼10 s of liters of subsurface fluid on to a 0.2-μm-mesh filter over the course of 80 min, prior to fixation in RNA Later. The SAG dataset was created from a ship-processed sample that was pumped into a bag that sat on the chassis of the ROV before being brought to the surface for processing approximately 10 h later. Regardless of these sample collection differences, the SAGs and MAGs capture similar populations of primarily Gammaproteobacteria, Bacteroidetes, Desulfobacterota, Alphaproteobacteria, and Zetaproteobacteria (Figure 1B and Supplementary Data Sheet 1). In comparison, taxonomy assessment from classification of the rRNA reads in the metatranscriptomic data suggests a less diverse community structure dominated by Gammaproteobacteria (Figure 1B). Similarly, mapping metatranscriptome reads to 16S rRNA genes in the SAG library shows that SAG AH-315-E17, a Gammaproteobacteria, recruits on average ∼46X more reads than any other 16S rRNA gene in any of the other SAGs (Figure 3 and Supplementary Data Sheet 5). Therefore, although the taxonomic composition between the SAGs and metatranscriptome looks different (Figure 1B), the read-mapping to 16S rRNA genes reveals there is more congruence than is apparent when comparing the composition of individual SAGs and metatranscriptome reads (Figures 1B, 3).

In addition to the natural variability of the crustal fluid microbial community observed at one location in a short window of time, the installation of the observatories at North Pond in 2011 also altered conditions within the subsurface which have taken several years to reset, although flow from one site to another can be quite rapid (Wheat et al., 2020). Observations of crustal fluid community structure at this site from 2012 and 2014 samples likely reflect a greater degree of seawater entrainment (i.e., Meyer et al., 2016; Tully et al., 2018), while observations in 2017 samples may be more reflective of pre-disturbance *in situ* conditions, as discussed elsewhere (Trembath-Reichert et al., 2021). Thus, the trends in DNA-based metagenomic read recruitment as a function of time (Figure 2) can be interpreted in the context of the microbial community returning to a natural state after drilling operations. The 2017 SAGs recruited more reads from the 2014 metagenomes than the 2012 metagenomes, and the most reads from the metagenomes sampled in 2017, as the initial pulse of seawater microbes leaves the system. By contrast, the more constant RNA recruitment statistics could indicate that taxa closely related to these SAGs may have been active throughout the sampling period, and that the entrained seawater microbiota was not active in the subsurface. This is consistent with lack of transcripts for surface-seawater-derived microbial taxa in the earlier years of sampling when the crustal system was still recovering from drilling-related disturbances (Seyler et al., 2020).

### Advantages of Single Cell Sorting for Low Biomass Samples

Metatranscriptomics is a challenging method for assessing activity due to microbial cellular RNA being mostly composed of stable ribosomal RNA (rRNA) with much less messenger RNA (mRNA). For example, metatranscriptomic studies of ocean surface-waters found that 52–88% of RNA libraries were comprised of rRNA (Stewart et al., 2010). A similar study found 44–56% percent rRNA in marine metatranscriptomes and calculated only 0.48–6.19 × 10^2^ copies of mRNA per cell in coastal seawater (Gifford et al., 2011). The challenges of this methodology are exacerbated when studying the deep biosphere where biomass is in the 10^2^–10^4^ cells ml^–1^ range (Tully et al., 2018; Seyler et al., 2020; Trembath-Reichert et al., 2021). A testament to these challenges is the low RNA yields (0.8–3.9 ng μl^–1^) from the North Pond aquifer that were too low for standard RNA-seq library preparation kits or the use of the rRNA depletion strategies applied to samples with greater biomass (He et al., 2010; Stewart et al., 2010; Seyler et al., 2020). Consistent with this, on average only 6.7% of the metatranscriptome libraries here are mRNA, and a large portion (93%) of the metatranscriptome library for the 2017 Hole U1383C Deep sample that mapped to the SAGs mapped to rRNA genes (Supplementary Data Sheet 5).

This pilot study suggests that single-cell sorting using redox active dyes could complement metatranscriptomic methods on low biomass samples because it does not rely on the quantity or stability of mRNA to gain information about the genome characteristics of cells. We have demonstrated that this method can be used on low-biomass samples using a fraction of the sample volume (10^–3^ liters compared 10^1^–10^3^ liters). This “activity based” single cell sorting approach is being validated further and is beyond the scope of this present pilot study. A limitation to the RedoxSensor*™* Green cell sorting-based approach is the need for ship-based incubation that may result in shifts in community structure or function if important *in situ* conditions cannot be replicated (i.e., temperature, pressure, oxygen). Sample transport to the ship and manipulation on board likely causes some changes to the microbial community and their activity (e.g., Fortunato et al., 2021; Trembath-Reichert et al., 2021). Regardless, for the North Pond oxic crustal fluids, our comparison reveals that single-cell sorting using a redox-active dye and paired metagenomics/metatranscriptomics identify many of the same taxonomic groups and metabolic functions, even when sorted cells were only sequenced at low coverage.

Deeper sequencing of SAGs would have undoubtedly led to the recovery of a greater fraction of their genomes (Stepanauskas et al., 2017). Subsequently, the same SAGs would have recruited more reads from the metagenomes and metatranscriptomes and recovered additional protein coding gene clusters. There is a linear relationship between increasing cumulative assembly length/number of protein-coding sequences and the number of gene-clusters (Figure 4). For example, adding the SAGs to the total MAG dataset increased the cumulative assembly length by ∼72% and increased the number of gene-clusters by ∼65% (Figure 3 and Supplementary Data Sheet 6). Studies of surface-ocean SAG data has shown that the number of functional gene-clusters does not begin to saturate until very high sequence depth (Pachiadaki et al., 2019; Duarte et al., 2020). The linear relationship between the number of gene-clusters as additional sequence information is added to the analysis suggests that the sequencing depth of neither the SAG nor the MAG datasets fully capture the functional diversity of this environment (Figure 3). Therefore, it is likely that with a moderate increase in sequencing depth, this collection of SAGs from 1 ml of crustal fluid could produce the same or greater amount of information as the entire MAG dataset. A caveat to this interpretation is that the analysis presented in Seyler et al. (2020) also includes gene-based un-binned sequence data that is not directly compared in this protein clustering workflow that only included SAGs and MAGs.

### Functions of the Microbial Community in Subsurface Crustal Fluids From North Pond

The interpretation of the metabolic capacity of North Pond crustal fluids from the single cell genome approach is in general congruence with prior studies (Meyer et al., 2016; Shah Walter et al., 2018; Tully et al., 2018; Seyler et al., 2020). With respect to carbon cycling, the SAGs encode genes for both heterotrophy as well as autotrophy powered by reducing sources including sulfur and hydrogen (Figure 5), although it is not possible to determine from SAG data alone if autotrophy or heterotrophy dominates in the system. This observation of genes for both autotrophic and heterotrophic modes of carbon cycling is consistent with recent stable-isotope based incubations that show roughly equivalent potential for carbon assimilation by heterotrophy and autotrophy (Trembath-Reichert et al., 2021). Mapping metatranscriptome reads from all years and locations show that these SAGs represent activate taxa with these functions, although the nature of the data only resulted in sparse mapping statistics (Figure 6).

Carbon fixation genes were observed in SAGs that also contain genes for the oxidation of sulfur compounds (Figure 5). For example, a Gammaproteobacteria (*Thiotrichaceae* SAG AH-315-C22) possesses genes for carbon fixation by the CBB cycle (rbcL, KO ID: K01601) and evidence of sulfur oxidation (soxZ, KO ID: K17227). The collection of *Desulfobulbia* SAGs that share ∼99% ANI with MAG NP171383CD-8 contained genes for autotrophy *via* the Wood-Ljungdahl pathway. SAG AH-315-G02 in this group contains a partial nitrogen fixation operon containing nifABDKH, of which a gene annotated as nifH (K02588) received mapped metatranscriptome reads (Figure 6). The genomes of these groups of low coverage SAGs did not contain the sulfur or hydrogen oxidation genes annotated in NP171383CD-8. These observations are consistent with genes for carbon fixation being located in MAGs with sulfur and nitrogen cycling genes (Seyler et al., 2020). Thus, the single cell sorting based on a redox-active dye provide additional evidence that sulfur and nitrogen cycling taxa are responsible for producing new carbon in this system.

Heterotrophy/mixotrophy may be conducted by numerous taxa, including Bacteroidetes, which are the most abundant Phylum in the sorted cells (Figure 1). Bacteroidetes are known to degrade recalcitrant marine dissolved organic matter (Fernández-Gómez et al., 2013; Krüger et al., 2019). For example, Bacteroidetes SAGs contain genes for beta-*N*-acetylhexosaminidase (KO Id: K01207) and diacetylchitobiose deacetylase (KO Id: K03478) (Supplementary Figure 2). Hole U1383C Deep crustal fluids have older and more refractory dissolved organic carbon compared to other shallower and younger horizons, and microbially-mediated oxidation is a likely mechanism for removal of organic carbon in the fluids (Shah Walter et al., 2018; Trembath-Reichert et al., 2021).

The low-coverage single cell genome approach could not resolve the potential activity of presumed iron cycling taxa in this system. While there were numerous low-coverage SAGs grouping within the Zetaproteobacteria, a taxon known for iron oxidation, none of these contained the cyc2 gene diagnostic for microaerophilic iron oxidation (McAllister et al., 2020a), although it was observed in the Seyler et al. (2020) dataset. The three clades of Zetaproteobacteria that were sorted are mainly comprised of SAGs, MAGs sampled from other horizons between 2012 and 2014 at North Pond, and isolate genomes from hydrothermal vent sites such as the Snakepit and Rainbow sites on the Mid-Atlantic Ridge, including the isolate genome of hydrogen-oxidizing *Ghiorsea* (Field et al., 2015; Mori et al., 2017; Tully et al., 2018; McAllister et al., 2020a; Supplementary Figure 3). SAG AH-315-E14 contains a putative nitrite reduction operon, of which a gene annotated as nitric oxide reductase (norB, K04561) received mapped metatranscriptome reads from the 2014 U1383CD library (Figure 6). Nitrite reduction by Zetaproteobacteria has been inferred from metagenomic data at the Lō’ihi seamount (Singer et al., 2013; McAllister et al., 2020b) and recently a nitrite reductase (NirK) homolog has been described in *Mariprofundus sp. EKF-M39* (Jain and Gralnick, 2020). Zetaproteobacteria were significantly enriched by nitrate amended treatments and lower-oxygen sites in biofilm mineral colonization experiments at North Pond (Orcutt B. N. et al., 2020). The Zetaproteobacteria SAGs also contain high oxygen-affinity *cbb*_3_—type cytochromes (Figure 5; Preisig et al., 1996). Cumulatively, this suggests that iron cycling may be linked to denitrification in the deeper, warmer, low-oxygen depths at North Pond (Shah Walter et al., 2018).

Several SAGs besides Zetaproteobacteria contained genes for denitrification (Figure 5). This is consistent with observations in Seyler et al. (2020) that denitrification and other (facultative) anaerobic processes were more abundant in the deeper, warmer, and less oxic Hole U1383C Deep horizon compared to shallower, cooler, and more oxic horizons. Nitrous oxide reductase (nosZ, K00376) appeared in Planctomycetes, Gemmatimonadetes and Bacteroidetes SAGs, with members of the Bacteroidetes also having nitric oxide reductase (norB, K04561). Microbe-mineral enrichment experiments at the shallow horizon of Hole U1383C identified several members of the Bacteroidetes that responded to low-oxygen and nitrate enrichment (Orcutt B. N. et al., 2020). Likewise, Seyler et al. (2020) identified Bacteroidetes as having roles in denitrification to ammonia and to nitrite.

Other nitrogen cycling pathways were also apparent in the activity-based SAGs. Consistent with prior studies of North Pond crustal fluids (i.e., Seyler et al., 2020), the activity-based SAGs contained taxa typically associated with ammonium oxidation (i.e., Thaumarchaeota, *n* = 1) and nitrite oxidation (i.e., Nitrospina, *n* = 1, and Nitrospira, *n* = 1; Figures 1, 3, 5). These low-coverage and incomplete SAGs did not contain any of the genes usually diagnostic for these processes, which is a caveat to the low-coverage SAG workflow. Ammonium oxidation genes were detected in low abundance in the U1383C Deep metatranscriptome sampled in 2017 (Seyler et al., 2020), which is consistent with prior observations from this site of Thaumarchaeota being a low-abundance taxa yet performing an important ecosystem function (Zhao et al., 2020). One instance of nitrogen fixation genes was detected in the activity-based SAGs, whereas this pathway was not detected in the metatranscriptome (Figure 5).

## Conclusion

Studying the ocean crustal subsurface entails unique methodological challenges. In this study we show that sorting of single cells using a redox-active dye from milliliters of crustal fluids has the potential to capture similar amounts of genetic information about microbes in comparison to metagenomic and metatranscriptomic workflows on much larger sample volumes. Due to the challenges of marine subsurface research, we conclude that this tool should be considered for future studies. Likewise, such low-volume approaches may also have relevance for sample collection strategies being considered for planetary rovers searching for life on other planets (Hendrix et al., 2019).

## Data Availability Statement

Flow cytometric data including index data are available at flowrepository.org using the MIFlowCyt guidelines (Lee et al., 2008; Spidlen et al., 2012). Single cell amplified genomes that were long enough for the NCBI assembly database are available under the BioProject accession number PRJNA702937. The total set of SAGs are also available here for download: https://figshare.com/articles/dataset/NorthPond_SAGs_tar_gz/14076425. Datasets that are used in comparison described in Seyler et al. (2020) are available at NCBI BioProject numbers PRJNA554681 (metagenomic reads) and PRJNA522799 (metatranscriptomic reads) and assembled MAGs are available here: https://figshare.com/articles/dataset/North_Pond_2017_High_Completion_Bins/12389789.

## Author Contributions

BO, NP, and RS designed the SAG experiment and secured funding. JH designed and secured funding for the comparison ‘omics datasets. TD’A, JG, and BO participated in field work to collect the samples. TD’A performed the experiment and designed and completed all bioinformatic analysis. JG and NP processed the samples. LS and JH performed the bulk omic analyses on companion samples. TD’A and BO wrote the manuscript with input from all co-authors. All authors contributed to the article and approved the submitted version.

## Conflict of Interest

The authors declare that the research was conducted in the absence of any commercial or financial relationships that could be construed as a potential conflict of interest.

## Publisher’s Note

All claims expressed in this article are solely those of the authors and do not necessarily represent those of their affiliated organizations, or those of the publisher, the editors and the reviewers. Any product that may be evaluated in this article, or claim that may be made by its manufacturer, is not guaranteed or endorsed by the publisher.

## Footnotes

1
https://github.com/Ecogenomics/BamM

## Supplementary Material

Supplementary Figure 1 Histogram of the assembly lengths [**(A)** in millions of base pairs, actual length of SAG/MAG assemblies, not complete genome length] and estimated genome completeness **(B)** for the 131 SAGs (this study) and 131 MAGs (from Seyler et al., 2020). In B, lines highlight the 90% (solid) and 50% (dashed) thresholds for the MIMAG High-Quality and Medium-Quality standards, respectively. All plotted assemblies contained < 5% contamination.

Supplementary Figure 2 KEGG Decoder output for SAG dataset.

Supplementary Figure 3 Phylogenetic reconstruction of Zetaproteobacteria from IMG including SAGs and MAGs from this study and Seyler et al. (2020). Bolded Red genomes include SAGs from this study, MAGs from Seyler et al. (2020) along with close relatives from other sites on the Mid Atlantic Ridge and the Loihi Seamount (McAllister et al., 2020a). Black bolded genomes are cultured members of the Zetaproteobacteria. NORP31 and NORP148 are MAGs from 2012 and 2014 North Pond crustal fluids (Tully et al., 2018). Collapsed clades are comprised of Zetaproteobacteria that are not relevant to these SAGs. The tree is rooted with a group of genomes from the Oceanospirillaceae family of the Gammaproteobacteria.

Supplementary Data Sheet 1 SAG assembly statistics as reported by the Single Cell Genomics Center at Bigelow Laboratory for Ocean Sciences.

Supplementary Data Sheet 2 Average Nucleotide Identity (ANI) statistics for the SAG library against the Hiqh-Quality MAGs from Seyler et al. (2020).

Supplementary Data Sheet 3 DNA metagenomic read recruitment (in Reads per Thousand Million, RPKM) for individual SAGs. This analysis includes metagenomic read samples from Tully et al., 2018 (samples starting with NP12 and NP14) and Seyler et al. (2020) (samples starting in NP17 and metatranscriptomic samples ending in -RNA).

Supplementary Data Sheet 4 Bulk DNA metagenomic read recruitment for the entire SAG library for the same samples used to calculate RPKM values for individual SAGs.

Supplementary Data Sheet 5 RNA metatranscriptomic read recruitment statistics to 16S genes in the SAG library.

Supplementary Data Sheet 6 Gene cluster statistics resulting from Anvio Pangenomics workflow.
