# Supplementary figures and images for: Oceanic Crustal Fluid Single Cell Genomics Complements Metagenomic and Metatranscriptomic Surveys With Orders of Magnitude Less Sample Volume
Source: Front Microbiol. 2022 Jan 24;12:738231. doi: 10.3389/fmicb.2021.738231 (PMC8819061; doi:10.3389/fmicb.2021.738231)

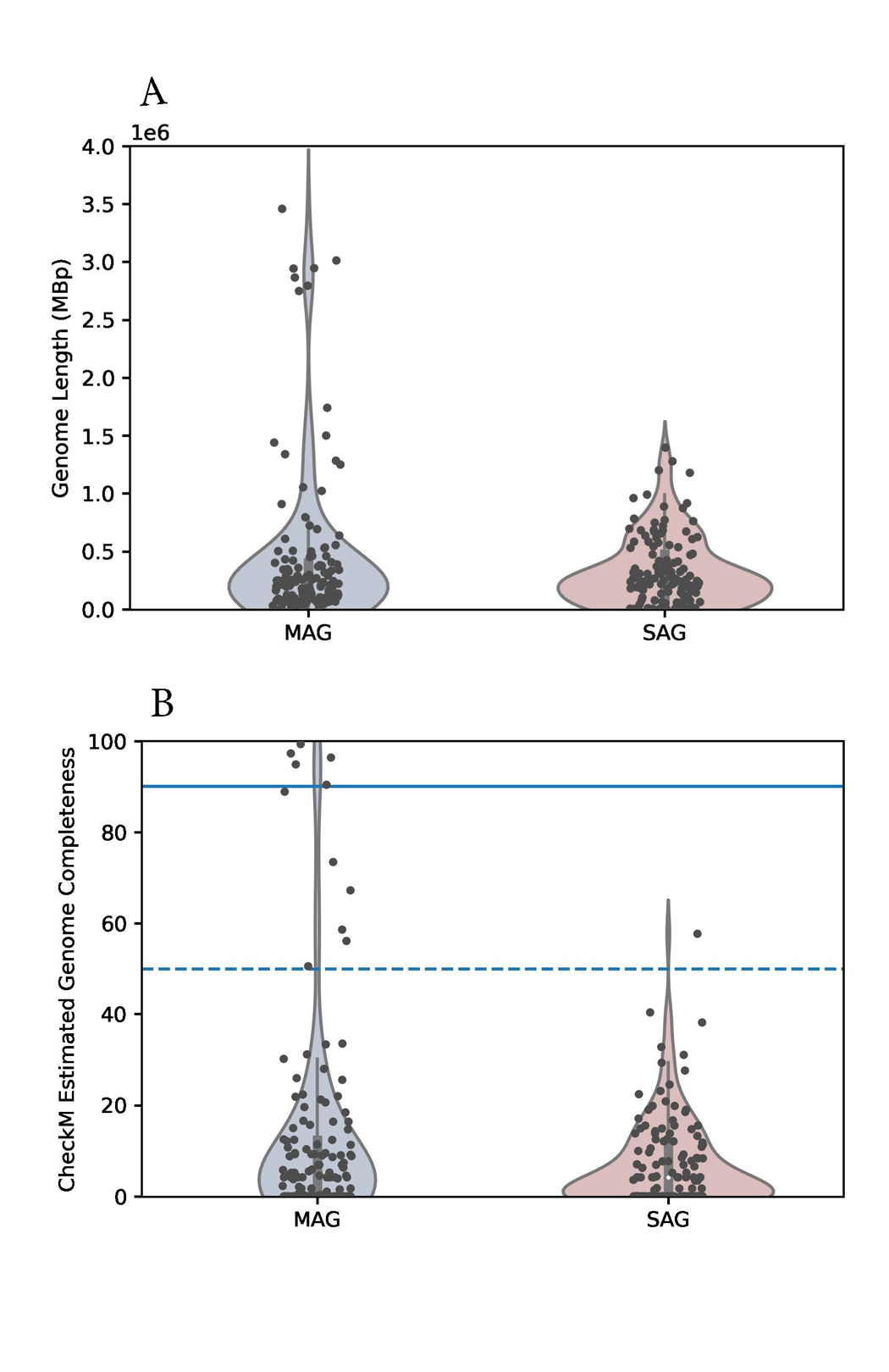

Supplement: Supplementary Figure 1 — Histogram of the assembly lengths [(A) in millions of base pairs, actual length of SAG/MAG assemblies, not complete genome length] and estimated genome completeness (B) for the 131 SAGs (this study) and 131 MAGs (from Seyler et al., 2020). In B, lines highlight the 90% (solid) and 50% (dashed) thresholds for the MIMAG High-Quality and Medium-Quality standards, respectively. All plotted assemblies contained < 5% contamination. [file Image_1.TIFF]

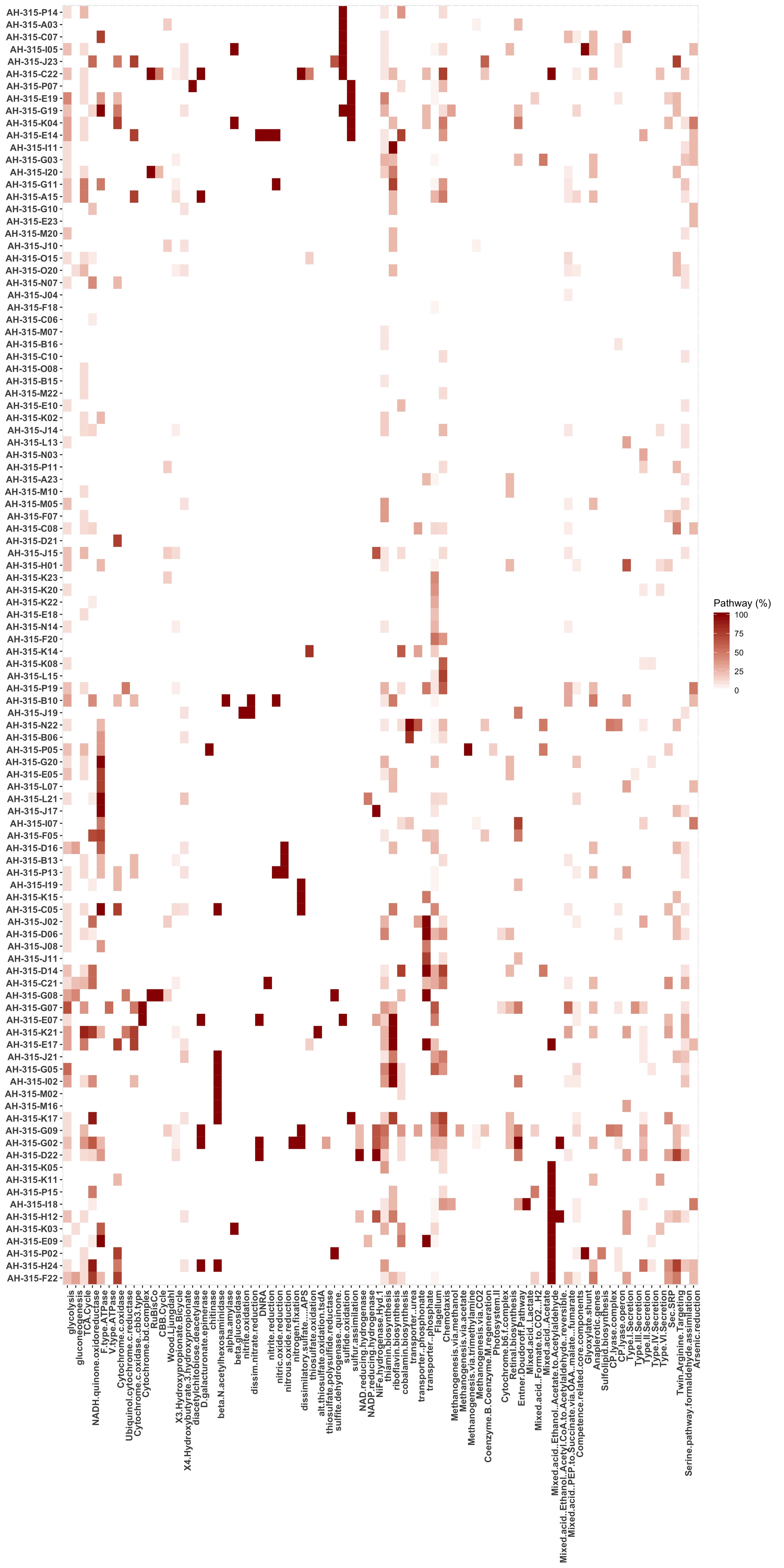

Supplement: Supplementary Figure 2 — KEGG Decoder output for SAG dataset. [file Image_2.JPEG]

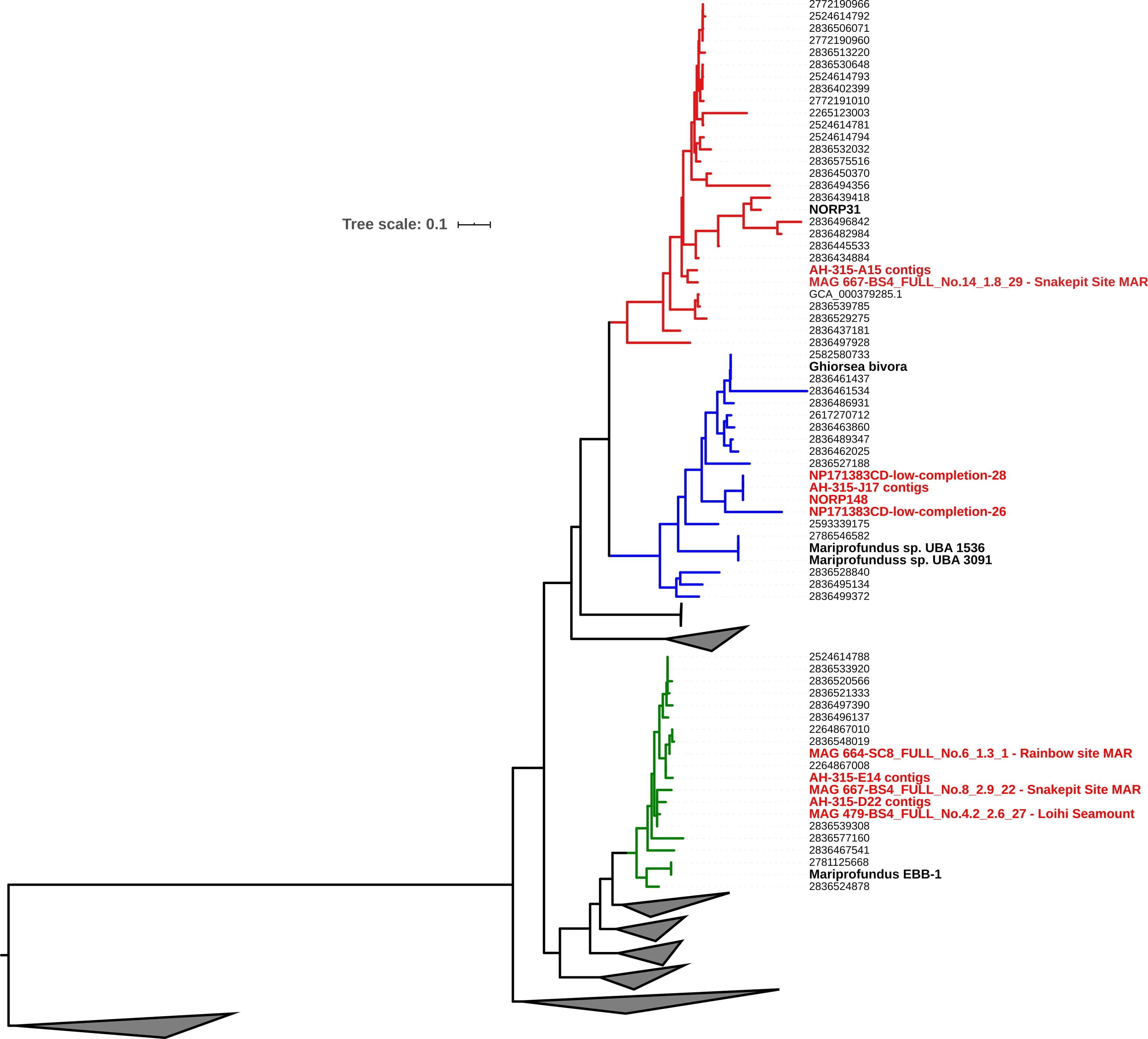

Supplement: Supplementary Figure 3 — Phylogenetic reconstruction of Zetaproteobacteria from IMG including SAGs and MAGs from this study and Seyler et al. (2020). Bolded Red genomes include SAGs from this study, MAGs from Seyler et al. (2020) along with close relatives from other sites on the Mid Atlantic Ridge and the Loihi Seamount (McAllister et al., 2020a). Black bolded genomes are cultured members of the Zetaproteobacteria. NORP31 and NORP148 are MAGs from 2012 and 2014 North Pond crustal fluids (Tully et al., 2018). Collapsed clades are comprised of Zetaproteobacteria that are not relevant to these SAGs. The tree is rooted with a group of genomes from the Oceanospirillaceae family of the Gammaproteobacteria. [file Image_3.JPEG]
